# The *NRP1* migraine risk variant shows evidence of association with menstrual migraine

**DOI:** 10.1186/s10194-018-0857-z

**Published:** 2018-04-18

**Authors:** Charmaine E. Pollock, Heidi G. Sutherland, Bridget H. Maher, Rodney A. Lea, Larisa M. Haupt, Alison Frith, E. Anne MacGregor, Lyn R. Griffiths

**Affiliations:** 1 Genomics Research Centre, Institute of Health and Biomedical Innovation, School of Biomedical Sciences, Queensland University of Technology, Brisbane, Australia; 2 Clinithink Limited, Bridgend, UK; 3 Centre for Neuroscience & Trauma, Blizard Institute of Cell and Molecular Science, London, UK

**Keywords:** Migraine, Menstrual migraine, Genome wide association study (GWAS), Neuropilin 1 gene (NRP1), Genetics, Single nucleotide polymorphisms

## Abstract

**Background:**

In 2016, a large meta-analysis brought the number of susceptibility loci for migraine to 38. While sub-type analysis for migraine without aura (MO) and migraine with aura (MA) found some loci showed specificity to MO, the study did not test the loci with respect to other subtypes of migraine. This study aimed to test the hypothesis that single nucleotide polymorphisms (SNPs) robustly associated with migraine are individually or collectively associated with menstrual migraine (MM).

**Methods:**

Genotyping of migraine susceptibility SNPs was conducted using the Agena MassARRAY platform on DNA samples from 235 women diagnosed with menstrual migraine as per International Classification for Headache Disorders II (ICHD-II) criteria and 140 controls. Alternative genotyping methods including restriction fragment length polymorphism, pyrosequencing and Sanger sequencing were used for validation. Statistical analysis was performed using PLINK and SPSS.

**Results:**

Genotypes of 34 SNPs were obtained and investigated for their potential association with menstrual migraine. Of these SNPs, rs2506142 located near the neuropilin 1 gene (*NRP1*), was found to be significantly associated with menstrual migraine (*p* = 0.003). Genomic risk scores were calculated for all 34 SNPs as well as a subset of 7 SNPs that were nearing individual significance. Overall, this analysis suggested these SNPs to be weakly predictive of MM, but of no prognostic or diagnostic value.

**Conclusions:**

Our results suggest that *NRP1* may be important in the etiology of MM. It also suggests some genetic commonality between common migraine subtypes (MA and MO) and MM. The identification of associated SNPs may be the starting point to a better understanding of how genetic factors may contribute to the menstrual migraine sub-type.

## Background

Migraine is a genetic disorder that affects more than 10% of the world’s population and is the third leading cause of disability for 15–49 year old women [1]. It is characterized by moderate to severe headache lasting up to 72 h often accompanied by nausea, photophobia, phonophobia and vomiting. The International Classification for Headache Disorders 3 (ICHD-3), from the International Headache Society, classifies migraine into two main subtypes: migraine without aura (MO) and migraine with aura (MA). MA has additional neurological symptoms preceding the headache including visual disturbances and sensory function impairment [2].

Epidemiological studies have shown that after puberty a distinct sex bias in migraine prevalence occurs and females are three times more likely to be affected by this disorder than males [3, 4]. It is reported that females have longer lasting and more severe migraine attacks than men [5, 6]. The evident conclusion is that there is a hormonal etiology for these migraines, particularly related to estrogen metabolic pathways [7, 8]. There is increasing evidence that the late luteal decline in estrogen is not only a trigger for migraine [9, 10], but potentially also the cause for the increase in pain severity [11]. In population- and clinic-based studies, between 20% and 60% of women with migraine report an association with menstruation [12]. This has led to a sub-classification of menstrual migraine (MM), classified in the ICHD-3 appendix for research purposes [2]. As per the ICHD-3 classification, MM is a sub-classification of MO, as menstruation does not appear to be associated with MA. Women suffering MM have reported symptoms to be more severe, prolonged and resistant to treatment than conventional migraine [13], and therefore is detrimental to quality of life.

Investigations into the estrogen and progesterone metabolic pathways have been undertaken to understand whether there is an underlying genetic basis for MM. However, there have been many contradictions and conflicting evidence regarding specific gene associations [14]. For example, estrogen metabolism genes *COMT*, *CYP1A1* and *CYP19A1* were found to have no significant association in an Australian cohort [8, 15], contradictory to earlier studies that found *COMT* to be statistically significant in research conducted in an American population [16]. *ESR1* polymorphisms have also been found to have an association with MM [15], and also with migraine not specifically related to menstruation [17]. *SYNE1* and *TNF* genes have been associated with MM, with these findings yet to be replicated in a larger population for further validation [15].

In 2016, Gormley et al. performed a meta-analysis of 375,000 individuals from 22 genome-wide association (GWA) studies and were able to identify 46 single-nucleotide polymorphisms (SNPs) significantly associated with migraine risk, which implicated 38 genomic loci [18]. Twenty eight of these loci had not been previously linked to migraine at the time of publication. Subtype analysis was also performed for both MO and MA, which showed that some SNPs were significantly associated with MO. However, no further analysis of other migraine subtypes, including MM, was conducted.

Using a case-control cohort in which the cases were specifically diagnosed with MM, this study aimed to test the hypothesis that SNPs robustly associated with migraine, as determined by Gormley et al. (2016), are also individually or collectively associated with MM.

## Methods

### Population cohort

A cohort of 268 females affected by MM, as well as 142 controls, were previously recruited by the City of London Migraine Clinic. Details of the demographics for the cohort are presented in Sutherland et al. [8]. The diagnosis for this classification included documented diary evidence over at least three consecutive menstrual cycles with migraine attacks occurring on day 1 ± 2 in at least two out of three of these cycles and/or at additional times of the cycle. The controls were females who had no personal or familial history of migraine and of a similar age. Biological samples were collected and transported to the Genomic Research Centre, Queensland, Australia. For this particular study DNA samples for 235 MM females and 140 controls were available for genotyping.

### Genotyping

AGENACX online MassARRAY® software was used to incorporate 40 of the 46 SNPs identified in the previous meta-analysis [18] into two multiplex assays. The software was used to design forward, reverse and extension primers for the SNPs to be assayed (sequences available on request).

PCR and extension primers were pooled and balanced according to the MassARRAY protocol (Agena Bioscience, San Diego, CA, USA). Extracted DNA from the MM population samples was diluted to a concentration of 20 ng/μl. Targeted loci were amplified using Taq polymerase, treated with Shrimp alkaline phosphatase (SAP) to dephosphorylate any unincorporated dNTPs, and an extension reaction was performed using iPLEX extension Gold reagents (Agena Bioscience, San Diego, CA, USA) according to the manufacturer’s protocol. Samples were subsequently desalinated with SpectroCLEAN® and the resulting products were spotted onto SpectroCHIPs using a Nanodispenser RS1000. Detection of primer extension products was performed by matrix-assisted laser desorption/ionization time-of-flight (MALDI-TOF) mass spectrometry. SpectroTYPER software was used to automatically import and analyze the genotyping data with genotypes called based on the calculated mass of the extension products.

Validation of MassARRAY results was undertaken using a number of alternative genotyping methods. For rs12845494 and rs2506142, restriction fragment length polymorphism (RFLP) assays were used. This involved amplification of the targeted loci using a standard PCR protocol followed by digestion with the restriction enzymes *Pst*I (NEB #R0140S) for rs12845494, and *Bsm*AI (NEB #R0529L) for rs2506142. The PCR product for rs12845494 was added to a master mix of *Pst*I enzyme with NEBuffer 3.1 and incubated at 37 °C for 16 h. For rs2056142, the PCR product was added to a master mix of *Bsm*AI with SmartCutter® and incubated for an hour at 55 °C. Products were subsequently run on 4% agarose gels for analysis.

For validation of genotypes for the SNP rs1024905 PCR-pyrosequencing was used. The primers 5’ TTTGGCCTCAGACCCCTTTA (biotinylated) and 5’ CATCAATGGATATAGCCCACATAA were used to amplify an 83 bp biotinylated PCR product and genotyping was performed on a QSeq pyrosequencer (Bio Molecular Systems). The biotinylated strand was immobilised on Steptavidin Mag Sepharose® beads (GE Healthcare) and the sequencing primer 5’TGGATATAGCCCACATAAG annealed prior to pyrosequencing using Pyromark® Gold reagents (QIAGEN). Sequencing traces were analyzed with QSeq software.

Sanger sequencing was used to validate genotype data for a subset of the SNPs (rs1024905, rs4910165, rs2506142), as minor allele frequencies deviated from that reported in databases. This was conducted using BigDye™ Terminator v3.1 Cycle Sequencing Kit (Applied Biosystems, Foster City, CA, USA) on the 3500 Genetic Analyzer (Applied Biosystems).

### Statistical analysis

SNPs with low call rates (< 80%) were excluded from further analysis. Statistical analysis was performed on a final set of 34 SNPs using PLINK V1.07 [19]. Case-Control association of SNPs to MM were analyzed in PLINK using the *--assoc* command.

A genomic risk score (GRS) was determined for each of the individuals included in this study using the --*score* command in PLINK V1.07 [19] and the average score calculated. This score was validated manually and the SNPs were weighted using the log odds ratios (OR) determined in the Gormley meta-analysis [18]. The significance threshold was set to *p* = 0.05, as this is a replication study. However, due to the number of SNPs tested, the Bonferroni correction for multiple testing value (*p* = 0.001) was also considered.

Analysis and comparison of genomic risk scores for case/control status was performed with logistic regression analysis and Nagelkerke’s pseudo *R*^*2.*^ Receiver operating characteristic (ROC) - area under the curve (AUC) was also calculated to investigate the classification (diagnostic) value. These analyses were performed using SPSS v23 (IBM Corp., Armonk, NY).

## Results

### Genotyping and quality control

Five of the 46 SNPs identified by Gormley et al. were excluded from the study (rs10218452, rs4814864, rs28455731, rs138556413 and rs12135062) due to difficulty in designing primers for genotyping the region of interest. To ensure only the inclusion of high quality data representative of our cohort, SNPs with a low call frequency (< 80%), as called by SpectroTYPER, were excluded from further analysis (rs140002913, rs11172055, rs67338227, rs186166891, rs12260159, rs111404218). From the original cohort 235 MM and 140 control individuals had DNA available for genotyping. Seven MM and nine control samples were eliminated from further analysis due to a low call frequency (< 90%). This cut off filter was applied to ensure we had complete confidence in the integrity of the sample DNA used in the final analysis. One of the 34 SNPs analyzed, rs1024905 (near FGF6), had a MAF that differed substantially from that annotated in databases and Gormley et al. (2016), and in some samples Sanger sequencing failed to confirm genotypes obtained from the MassARRAY assay (rs4910165). Therefore pyrosequencing was used as an alternative method of genotyping some SNPs. This resulted in statistical analysis being performed with 228 case samples, 131 control samples and 34 SNPs.

### Association analysis

As this study is a replication of the results obtained by Gormley et al. (2016), a significance cut-off of *p* < 0.05 was initially applied. Analysis of the genotyping data for each of the 34 SNPs is presented in Table 1 and revealed a significant association for SNP rs2506142 in the *NRP1* locus with MM (*p* = 0.003). MassARRAY genotyping results for this SNP were confirmed using an RFLP assay, as well as through Sanger sequencing validation of a subset of samples. No other SNPs were significantly associated with MM, although a number including rs11624776 (*p* = 0.098), rs6724624 (*p* = 0.081), rs1925950 (*p* = 0.092), rs6693567 (*p* = 0.060), rs6791480 (*p* = 0.072) and rs111172113 (*p* = 0.074) had *p*-values that were approaching the *p* < 0.05 threshold. However, it should be noted that if a Bonferroni correction is applied for testing multiple SNPs (*p* < 0.0015), none of the 34 SNPs assayed were significantly associated with MM.

**Table 1:** Summary of the results obtained for the 34 SNPs from Gormley et al. genotyped in the menstrual migraine population

Locus	CHS	Index SNP	Minor allele	MAF controls	MAF cases	OR (95% CI)	*p*-value
*LRP1*-*STAT6*-*SDR9C7*	12	rs11172113	C	0.3571		0.7312 (0.5183–1.032)	0.0742
*FHL5*-*URF1*	6	rs4839827	T	0.497	0.4879	0.9098 (0.6671–1.241)	0.5506
		rs7775721	T	0.3779	0.4013	1.104 (0.8068–1.51)	0.5367
Near *TSPAN2*-*NGF*	1	rs2078371	C	0.1192	0.1239	1.045 (0.6542–1.668)	0.8549
		rs7544256	A	0.3893	0.3778	0.9405 (0.6874–1.287)	0.7012
*TRPM8*-*HJURP*	2	rs10166942	C	0.2045	0.1806	0.8609 (0.5858–1.265)	0.4456
		rs566529	G	0.1789	0.1401	0.748 (0.4816–1.162)	0.1954
		rs6724624	C	0.2365	0.2589	1.39 (0.9593–2.013)	0.08106
*PHACTR1*	6	rs9349379	G	0.3821	0.3523	0.8804 (0.6365–1.1218)	0.4415
*MEF2D*	1	rs1925950	G	0.3566	0.4189	1.31 (0.9557–1.797)	0.09287
Near *FGF6*	12	rs1024905	G	0.4511	0.4762	0.904 (0.6636–1.232)	0.5225
*PLCE*	10	rs10786156	G	0.4665	0.466	0.9669 (0.7011–1.333)	0.8372
		rs75473620	T	0.05479	0.05523	1.036 (0.5016–2.141)	0.9235
*KCNK5*	6	rs10456100	T	0.2863	0.2851	0.9943 (0.7103–1.392)	0.9733
*ASTN2*	9	rs6478241	A	0.3575	0.3649	1.091 (0.7911–1.504)	0.5958
*CFDP1*	16	rs8046696	T	0.4476	0.4552	1.087 (0.7987–1.4979_	0.5965
*RNF213*	17	rs17857135	C	0.171	0.1689	0.9623 (0.6404–1.446)	0.8532
*NRP1*	10	rs2506142	G	0.1163	0.2054	1.965 (1.244–3.102	0.00335*
Near *GPR149*	3	rs13078967	C	0.01705	0.01802	1.174 (0.3501–3.938)	0.7945
Near *REST*-*SPINK2*	4	rs7684253	C	0.4887	0.4667	0.8045 (0.5924–1.093)	0.1633
Near *ZCCHC14*	16	rs4081947	G	0.3809	0.3927	1.133 (0.817–1.572)	0.4537
*HEY2*-*NCOA7*	6	rs1268083	C	0.4957	0.5158	1.212 (0.8921–1.646)	0.2187
Near *WSCD1*-*NRP1*	17	rs75213074	T	0.0178	0.02821	0.7476 (0.2256–2.477)	0.633
Near *TGFBR2*	3	rs6791480	T	0.3218	0.352	1.395 (0.9702–2.007)	0.07192
Near *ITPK1*	14	rs11624776	C	0.3617	0.3399	0.7676 (0.5605–1.051)	0.0987
Near *ADAMTSL4*-*ECM1*	1	rs6693567	C	0.2914	0.3168	1.425 (0.9848–2.062)	0.05964
Near *CCM2L*-*HCK*	20	rs144017103	T	0.02821	0.02821	1.013 (0.3873–2.649)	0.9792
*YAP1*	11	rs10895275	A	0.3288	0.3309	1.02 (0.7056–1.475)	0.9149
Near *MED14*-*USP9X*	X	rs12845494	G	0.209	0.2441	1.222 (0.8362–1.787)	0.2996
Near *DOCK4*-*IMMP2L*	7	rs10155855	T	0.05241	0.04933	0.8475 (0.4316–1.664)	0.6305
1p31.1	1	rs1572668	G	0.4677	0.4948	1.114 (0.8095–1.534)	0.5064
*ARMS2*-*HTRA1*	10	rs2223089	C	0.09218	0.08772	0.8654 (0.515–1.454)	0.5849
*IGSF9B*	11	rs561561	T	0.105	0.1123	1.244 (0.7465–2.075)	0.401
*MPPED2*	11	rs11031122	C	0.2247	0.2222	0.9619 (0.6685–1.384)	0.8343

*CHS* chromosome *Homo sapiens*, *SNP* single nucleotide polymorphism, *MAF* minor allele frequency, *OR* odds ratio, *CI* confidence interval

*The SNP located near *NRP1* (rs2506142) is significant at *p*-value < 0.05

### Genomic risk score (GRS) analysis

**Fig. 1:**
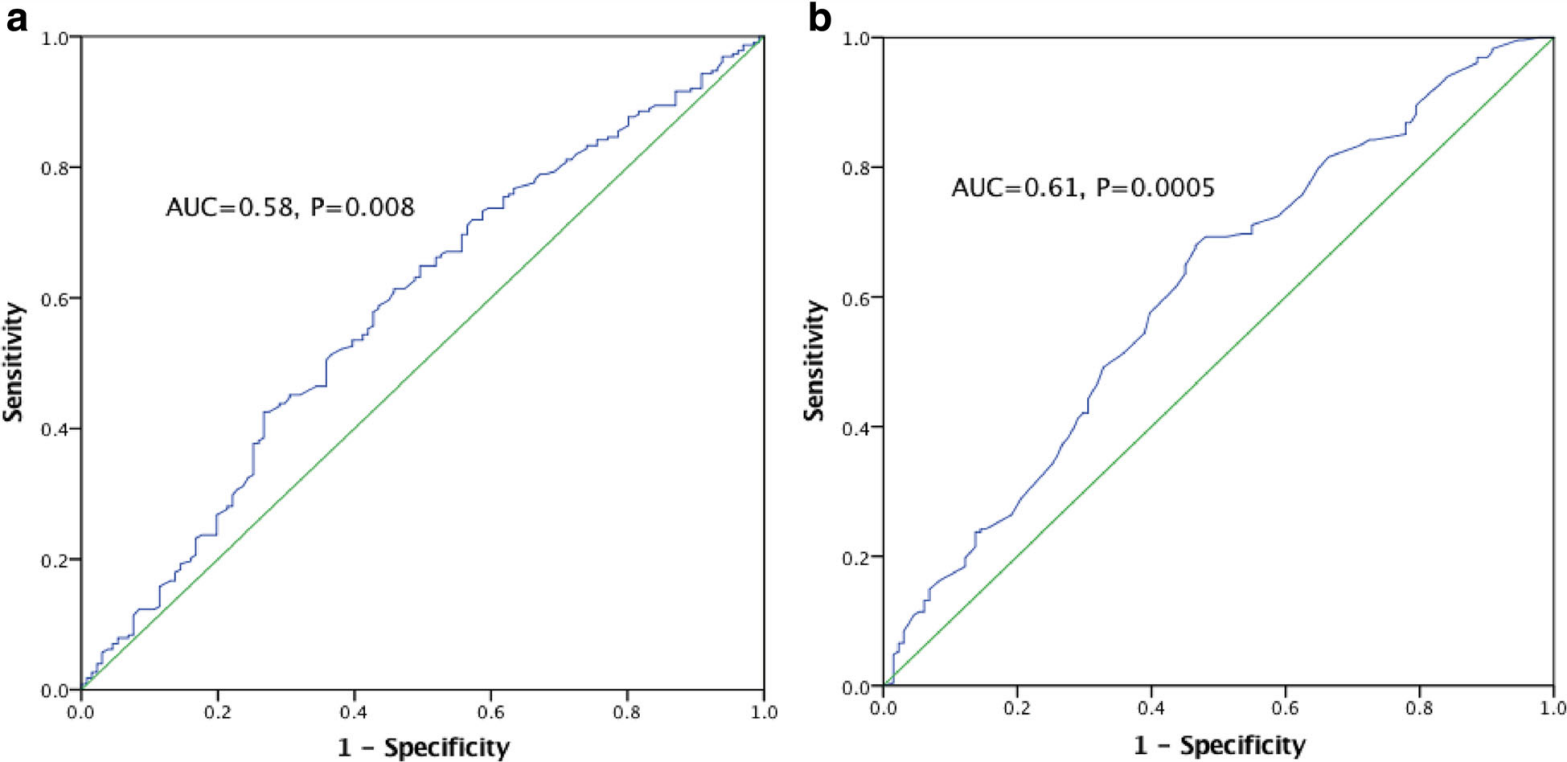
AUC for the MM cohort considering all genotype SNPs (**a**) and only those SNPs that individual exhibit a nominal level of significance (**b**)

In order to determine any commonality in the genetic basis of MM to that of MA/MO as identified by Gormley et al. [18], we undertook GRS analysis of our 34 genotyped SNPs to investigate if they would show a signal not apparent at an individual SNP level. The GRS analysis revealed a small difference between the case population (Mean = 38.6, SD = 3.7) and control samples (Mean = 37.4, SD = 3.7). This difference, although suggestively significant, is very modest in terms of increased risk of MM (OR = 1.08, *p* = 0.015). Logistic regression analysis results were also significantly predictive, though low (*R*^*2*^ = 0.023, 95% CI = 1.014–1.14). The ROC-AUC analysis was also very weakly predictive (AUC = 0.58, *P* = 0.008) (Fig. 1a).

As the composite association of *all* genotyped SNPs proved to be quite small, GRS analysis was also conducted using the subset of SNPs that were or approaching a nominally significant value (*p* < 0.1), which comprised of rs2506142, rs11624776, rs6724624, rs1925950, rs6693567, rs6791480 and rs111172113. This selective approach resulted in a slightly greater predictive value (AUC = 0.61, *p* = 0.0005) (Fig. 1b).

## Discussion

The first GWA study in relation to migraine was conducted in 2010 and identified the first significant GWAS association [20]. In 2011, three more susceptibility loci were identified in migraine, with further categorization into MA and MO subtypes [21]. Although this study was performed using female migraineurs from the Women’s Genome Health Study, association testing for the MM subtype was not conducted. A small number of GWA studies have been performed attempting to identify SNPs associated with the MO and MA subtypes [22], or within an isolated population [23], but at time of writing, there have been no GWA studies specifically analyzing MM. In this study, we tested the association of previously identified migraine-related SNPs in a specific MM cohort and found evidence for a potential role of rs2506142 in the Neuropilin-1 (*NRP1*) gene at 10p11.22 (OMIM: 602069).

*NRP1* encodes a transmembrane protein that acts as a receptor for class 3 semaphorins, molecules that act to guide neuronal development via repulsive axon guidance in nervous and vascular systems [24]. Studies using knock-out mice with null or mutated *Nrp1* showed dysmorphic development of axons and the spinal cord in developing embryos [25]. Interestingly, in another study, angiogenesis and arteriogenesis were revealed to be compromised in the postnatal heart and retina [26]. Studies have also found that NRP1 is also expressed in smooth muscle cells and may be involved with contractility and mobility of cells within the gastrointestinal system [27]. Therefore, NRP1 functions could be related to either the neuronal or vascular aspects of migraine pathophysiology.

The actions of NRP1 are also involved in menstruation. The endometrium undergoes growth, remodeling and shedding of vasculature during the menstrual cycle as a response to the ovarian steroids estrogen and progesterone. An increase in estrogen is seen during the endometrium’s proliferative state, while progesterone is associated with the secretory phase, where maturation and remodeling of the vascular tissue occurs [28, 29]. Endometrial repair is facilitated by angiogenesis and is initiated following the withdrawal of ovarian hormones. Repair of the endometrial vasculature begins while menstruation is still in progress, meaning the vascular breakdown and repair is occurring concurrently within the endometrium [28, 29]. The hormones act on these systems via the key regulators, Vascular Endothelial Growth Factors (VEGFs) and their co-receptors from the semophorin family, including NRP1. In particular, estrogen regulates VEGF-A expression, resulting in angiogenesis during the proliferative phase of endometrial remodeling [28, 30].

Given the involvement of NRP1 in pathways of neurovascular tissue and menstruation, this transmembrane protein could very well play a role in the pathophysiology and etiology of MM. There is limited research regarding the expression of NRP1 specifically during menstruation but an increase in activity has been shown during the proliferative phase of endometrial remodeling [31], which correlates with the drop in estrogen that is also believed to trigger MM. Further investigation may reveal the correlation between this variant SNP and the associated risk with MM.

GRS analysis was performed to ascertain whether a composite of the 34 SNPs could be used to predict MM in an individual sample. The overall predictive value of MM risk based on these SNPs was low (*p* = 0.008). GRS analysis using only the 7 SNPs that were of suggestively significant value (*p* < 0.1) yielded a slightly more significant result (*p* = 0.0005) with an AUC of 0.61, but would still not be of diagnostic or prognostic value, as an AUC of 0.7 and above is required for a test of good to excellent diagnostic accuracy [32]. The GRS also did not confer an OR that was greater than any of the SNPs individually investigated. Overall these results provide evidence that while MM and other phenotypes of migraine may have some genetic commonality, including the rs2506142 *NRP1* SNP, there may be distinct genetic differences. For example, given the increase in activity of NRP1 during menstruation, it might be that this marker is more predictive of menstrual migraine occurring during menstruation in association with endometrial prostaglandin release rather than estrogen ‘withdrawal’ [12]. Given that management of MM is currently empirical, a specific marker that could enable more targeted treatment has obvious clinical benefits.

The Gormley et al. meta-analysis did not distinguish sub-classifications other than MA and MO, and included some self-reported migraine by questionnaire, so it is likely that menstrual-migraineurs constituted a proportion of the MO population. Due to the difficulties in gaining a rigorous assessment of migraine in relation to menstruation timing, these group distinctions are lost in large cohort studies. Therefore, some overlap of significant SNPs may be expected between the study by Gormley et al. and our studies. However, a limitation of our study was the small population size, particularly as the effect sizes of migraine susceptibility SNPs identified to date are small [18], potentially resulting in false-negative results. Furthermore, if a Bonferroni correction for multiple testing is applied, none of the SNPs would be individually significantly associated with MM. Nevertheless, further investigation of rs2506142, as well as the other SNPs, in a larger MM population is warranted. Obtaining a large enough sample size for discovery of MM-specific SNPs is likely to be difficult, as suggested by the lack of MA-specific SNPs found by Gormley et al. [18]. However, replication-type studies, without the need to correct so stringently for multiple testing, may prove to be informative.

## Conclusion

Migraine is the third leading cause of disability for women of reproductive age, with a significant burden on quality of life. While recent GWAS have increased the understanding of the genetic basis of migraine, the etiologies of some of the subtypes, including MM is largely unknown and targeted research is required. Our study suggests that *NRP1* may play a particular role in MM, however, replication in a larger MM cohort would be necessary to confirm this. Calculation of a GRS score for the genotyped migraine susceptibility SNPs in the MM cohort indicates some genetic commonality between migraine (MA and MO) and MM, but is not diagnostically useful.

## Abbreviations

AUC: Area under curve
GRS: Genomic Risk Score
GWA: Genome-wide association
ICHD-3: The International Classification of Headache Disorders
MA: Migraine with Aura
MALDI-TOF: Matrix-assisted laser desorption/ionization time-of-flight
MM: Menstrual Migraine
MO: Migraine without Aura
NRP1: Neuropilin-1
OR: Odds Ratio
RFLP: Restriction fragment length polymorphism
ROC: Receiver operating characteristic
SAP: Shrimp alkaline phosphatase
SD: standard deviation
SNP: Single-nucleotide polymorphism
VEGF: Vascular Endothelial Growth Factor
